# The relationship between pelvic organ prolapse and short birth intervals in a rural area of Nepal

**DOI:** 10.1186/s41182-021-00298-z

**Published:** 2021-01-15

**Authors:** Rupa Singh, Sandeep Mahat, Sonam Singh, Carolyn K. Nyamasege, Yukiko Wagatsuma

**Affiliations:** 1 Graduate School of Comprehensive Human Sciences, University of Tsukuba, 1-1-1 Tennodai, Tsukuba, Ibaraki 305-8575 Japan; 2 Resident Medical Officer, Department of Radiology, Bir Hospital, Kathmandu, Nepal; 3 Medical Officer at Birendra Sainik Hospital, Chauni, Kathmandu, Nepal; 4 Faculty of Medicine, Department of Clinical Trials and Clinical Epidemiology, University of Tsukuba, 1-1-1 Tennodai, Tsukuba, Ibaraki 305-8575 Japan

**Keywords:** Reproductive health, Pelvic organ prolapse, Birth interval, Nepal

## Abstract

**Background:**

Pelvic organ prolapse (POP) is one of the main contributors to reproductive health problems that affect women’s quality of life. Previous studies have reported the risk factors and prevalence of POP. The aim of this study is to examine the association between POP and short birth intervals in a rural area of Nepal.

**Methods:**

A cross-sectional study was conducted in Panchapuri municipality, located in Surkhet District of Karnali Province in Nepal. A questionnaire was used to collect information on POP, birth intervals, and other known confounding factors, such as age and parity. Multiple logistic regression analysis was used to examine the association between minimum birth intervals and POP.

**Results:**

The study recruited 131 women. The prevalence of POP was 29.8%. The mean (SD) of maternal age was 32.3 (0.7) years. The median parity was 2, with a range of 2–6 children. More than half (64.9%) of the women reported a minimum birth interval of less than 2 years. Maternal age at birth, minimum birth interval, parity, and latest birth interval were significantly associated with POP in univariate analyses. After adjusting for the potential confounding factors such as age and occupation, the minimum birth interval was significantly associated with POP [AOR = 3.08, 95% CI 1.04–9.19].

**Conclusion:**

The prevalence of POP was high in this rural area of Nepal. Age, parity, occupation, and minimum birth interval were significantly associated with POP.

**Supplementary Information:**

The online version contains supplementary material available at 10.1186/s41182-021-00298-z.

## Background

Pelvic organ prolapses (POP) is the bulging or protrusion of the pelvic organs, including the bladder, rectum, small bowel, and uterus and their associated vaginal segments, into or through the vagina [1]. It is a disorder commonly seen in older women. The prevalence of POP has been reported as 9% in the world in the global burden of diseases study in 2012 [2]. Whereas other studies from various countries reported the ranges from 2.9 to 41.1% [3–6]. It is difficult to obtain consistent prevalence statistics because POP diagnosis (based on symptoms, physical examination, or surgery) differs across studies [5, 6].

While POP is generally considered a postmenopausal disease, considerable attention has been given to the disease burden among women of reproductive age in less developed countries [7, 8]. One study reported a POP prevalence rate of 37% in Nepal [9]. The western region of Nepal, including Panchapuri Province, reported to have a higher risk of POP [9]. In low-income countries, women are prone to believe or are made to believe that reproductive health problems such as falling of the uterus are simply a woman’s fate [10, 11]. Poor reproductive health among women is a major public health problem in many developing countries, including Nepal. Delivering at home for the first-time pregnancy is another obstetric factor associated with an increased risk of POP. Besides, home delivery in a low-income setting is also linked to an increased risk of poor maternal and perinatal outcomes [10].

Closely spaced deliveries and multiple births may have long-term implications for women’s reproductive health [12]. The more vaginal births women have, the greater the possibility of POP [13]. Short birth intervals are still prevalent and mostly unplanned in the most part of the world [14]. In Nepal, 21% of child is born less than 2 years of interval. However, it differs by zone and region [15]. Those who have suffered a pregnancy or child loss are more likely to replace that pregnancy/child and hence the interval between births is short. Knowledge of birth spacing and its underlying characteristics, such as illiteracy, early marriage, lack of family planning, and poverty, are relevant to understanding reproductive patterns and fertility behavior [16]. Epidemiological studies of POP incidence and remission are scarce [17]. Valid information on the prevalence of POP is crucial for gynecological and reproductive health care planning [4].

Many researchers reported about the relationship between birth spacing and maternal and child health [12, 18]. Women with short birth intervals are associated with higher maternal mortality [19]. However, limited studies have been done on the relationship between short birth interval and the occurrence of POP [20, 21]. Therefore, preventive efforts can be suggested if the interval between each birth relates to the occurrence of POP. This study aimed to examine the association of short birth intervals of less than 2 years and the occurrence of POP among reproductive age women in a rural area of Nepal.

## Methods

### Study area and population

This study was conducted in Panchapuri municipality, located in Surkhet District of Karnali Province in Nepal. Surkhet is located nearly 580 km west of Kathmandu, the capital city of Nepal. The total population is 32,231, and the municipality covers an area of 329.9 km^2^ [22]. This area was chosen because of its high prevalence of POP [9, 11]. Panchapuri municipality has only one primary health care centers (PHCCs), called Salkot, and 4 other health posts. Data collection was conducted in five villages where PHCCs and health posts exist. The data collection period was from February to April 2019. Married women aged between 18 and 49 with a history of at least two deliveries were asked to participate in the study. Approximately equal numbers of women were recruited from each of the four health post catchment areas. If hysterectomy was conducted due to POP, such cases were also included. The woman who did not sign the consent form was excluded.

### Study design

A cross-sectional study was conducted. A semi-structured questionnaire was used to collect the data through a face-to-face interview. Eight interviewers, including two staff nurses and six female community health volunteers (FCHVs), assisted with the data collection. The interviewers were trained on the procedures for administering the questionnaire and on the symptoms and treatment of POP.

### Information included in the questionnaire

The study participants may not be well informed on the medical manifestations of POP. Therefore, the interviewer used a language that would be understandable to the lay person when asking questions, e.g., “Do you feel a dragging lump coming down in or outside the vagina?” Women’s recognition of the presence of this condition was considered the presence of POP. Socio-economic and demographic information of the households and caregivers were also collected. The questionnaire for this study was developed in reference to a standardized questionnaire regarding POP signs, symptoms, risk factors, treatment options, and preventive measures [23]. The Prolapse Quality of Life Questionnaire (P- QOL) was used to evaluate POP symptoms and quality of life [24]. Moreover, the tools were modified after being translated into Nepali language and were pretested in Surkhet district of Nepal before use by trained interviewers. Socio-economic and demographic information on the households was included in section I, whereas questions related to POP and health service-related information were included in sections II and III (supplementary material S1).

### Narrative study of self-reported reasons for POP

We conducted a narrative study was conducted by interviewing the participants regarding their self-reported reasons for POP. Women used many different words to describe POP. The WHO subgroup sample questions such as “do you feel anything coming out of your vagina?”, “Do you have a feeling of heaviness?”, and “is it uncomfortable down below?” were asked to elicit the presence of POP. These questions from the WHO can identify 80–90% of moderate and severe POP. The study subjects were encouraged to freely share their experiences related to reproductive issues during and after pregnancy. Most common feedbacks from mothers were selected and reported in the results. The interviewers were asked to take more detailed notes when there was a POP case. Voices were recorded and used later for the qualitative study. For almost all cases, women spent about 30–40 min giving a more in-depth explanation.

### Sample size calculation

We calculated the sample size based on testing the hypothesis of the differences in POP prevalence between the group with a birth interval of < 2 years and the group with a birth interval of ≥ 2 years. It was assumed that the group with a birth interval of < 2 years has a POP prevalence of 30% from the previous study [25] while the other group a prevalence of 15% [26]. The required sample size was calculated as 126, with a 5% attrition rate to account for missing data, a power of 0.80, and an alpha value of 0.05 based on a 2-sided test [27].

### Study variables

Minimum birth interval and immediately preceding birth interval were the three predictor variables used in this study while POP prevalence was the outcome. Age, parity, occupation, and education were used as co-variates as guided by existing literatures [8, 9]. The minimum birth interval was defined as the minimum length (years) in any birth intervals from each woman. A cutoff value of 2 years was used since WHO recommends a healthy pregnancy interval of at least 2 years (24 months), whereas immediately preceding birth interval was defined as the most recent birth interval.

### Statistical analysis

Descriptive analysis was performed to determine the frequencies and percentages of the characteristics of the study participants (education, occupation, ethnicity, monthly income, age at first childbirth, contraceptive use, place of delivery). Age and parity were analyzed as categorical variables. Education was categorized as none, primary, and secondary and above. Occupation was also categorized into three groups: business, commercial farm, and housewife. Univariate logistic regression was used to test the association between the selected variables and POP. Multivariate logistic regression analysis was then performed to examine the association between POP and the minimum birth interval with the adjustment of co-variates. Multicollinearity of the variables included in the analysis was examined. A *p* value of < 0.05 was considered statistically significant. All analyses were conducted with IBM SPSS version 24. Codes and themes were not created for qualitative data because of the limited responses, only from a few of the women presenting with POP. Consequently, we reported some of the narratives from the women presenting with POP.

### Ethical considerations

This study was reviewed and approved by the Ethical Committee of the Faculty of Medicine, University of Tsukuba, and the Nepal Health Research Council (NHRC). To minimize discomfort and inconvenience, the survey team explained the objectives of the study in a nonthreatening and culturally relevant manner. The participants were given an opportunity to ask questions and to decline participation freely. Women provided a written informed consent were enrolled in the study. Collected data was verified and entered into a secure database with restricted access to maintain confidentiality.

## Results

A total of 131 mothers from various village development committees (VDCs) in Panchapuri municipality in Surkhet, western Nepal, were interviewed. The general characteristics of the study participants are presented in Table 1. The mean (SD) maternal age was 32.3 (0.7) years. The median parity was 2, with a range of 2–6 children. There were no women aged below 20 years even though we planned to interview the targeted age group of 18 to 49. Hence, the age was categorized above 20 years old in Table 1, 2, and 3. The majority (65.6%) of the women had never been to school. Only 22.9% of the women had secondary and above level of education. More than half of the participants (51.9%) had at least 2 children. A short birth interval of less than 2 years was reported by 64.9% of the women. Approximately half of the women (52.7%) had worked more than usual during pregnancy, while 16.8% had worked less. Moreover, 89.3% of them stated that they visit public health facilities for treatment. Among those with POP, 76.9% were satisfied with the prolapse treatment that they received at health facilities.

**Table 1:** General characteristics of the study subjects (*N* = 131)

Characteristics	*N*	%
**Age, years** (mean ± SD)	32.27 ± 6.91	
**Education**		
None	86	65.6
Primary	15	11.5
Secondary and above	30	22.9
**Occupation**		
Business	48	36.6
Commercial farm	49	37.4
Housewife	34	26.0
**Ethnicity**		
Brahmin	35	26.7
Chhetri	25	19.1
Dalit	63	48.1
Other	8	6.1
**Monthly income, Nepalese. Rs.**		
> 5000	21	16.0
5000-	28	21.4
10000-	19	14.5
15000-	21	16.0
≥ 20000	42	32.1
**Age at first childbirth, years**		
≥ 20	101	77.1
< 20	30	22.9
**Contraceptive use**		
Yes	76	58.0
No	55	42.0
**Place of delivery**		
Home	61	46.6
Health facility	70	53.4

**Table 2:** Univariate logistic regression analysis for pelvic organ prolapse (*N* = 131)

Variables	POP *n* (%)	Non-POP *n* (%)	OR [95% CI]^a^	*p* value
**Minimum birth interval**				
≥ 2 years	7 (15.2)	39 (84.8)	Ref.	
< 2 years	32 (37.6)	53 (62.4)	3.36 [1.35, 8.41]	0.009
**Immediately preceding birth interval**				
≥ 2 years	12 (18.8)	52(81.2)	Ref.	
< 2 years	27 (40.3)	40(59.7)	2.93 [1.32, 6.48]	0.008
**Age, years**				
20–29	3 (6.1)	46 (93.9)	Ref.	
30–39	19 (34.5)	36 (65.5)	8.09 [2.22, 29.49]	0.002
40–49	17 (63.0)	10 (37.0)	26.07 [6.39, 106.24]	< 0.001
**Parity**				
2	10 (25.6)	58 (63)	Ref.	
3	11 (28.2)	28 (30.4)	2.28 [0.87, 5.99]	0.095
≥ 4	18 (46.2)	6 (6.6)	17.40 [5.55, 54.51]	< 0.001
**Occupation**				
Business	9 (23.1)	39 (42.4)	Ref.	0.494
Commercial farm	12 (30.8)	37 (40.2)	1.41 [0.53, 3.72]	0.002
Housewife	18 (46.1)	16 (17.4)	4.88 [1.81, 13.11]	

^a^*OR = [95% CI]* Odds ratio, *CI* Confidence interval, *Ref.* Reference category

**Table 3:** Multivariable logistic regression analysis for pelvic organ prolapse (*N* = 131)

Variable categories	AOR [95% CI]^a^	*p* value
**Minimum birth interval**		
≥ 2 years	Ref.	
< 2 years	3.08 [1.04, 9.19]	0.043
**Occupation**		
Business	Ref.	
Commercial farm	1.31 [0.43, 3.99]	0.632
Housewife	4.04 [1.27, 12.8]	0.018
**Age, years**		
20–29	Ref.	
30–39	6.77 [1.79,25.66]	0.005
40–49	15.60 [3.61, 67.41]	< 0.001

^a^*AOR [95% CI]* Adjusted odds ratio [95% confidence interval], *Ref.* Reference category

Table 2 shows the univariate analysis for POP with selected variables. The minimum birth interval less than 2 years showed higher prevalence of POP (OR = 3.36, 95% CI 1.35–8.41). Moreover, immediately preceding birth interval had also a significant relationship with POP. Women with the latest interval of less than 2 years were almost 3 times more likely to experience POP than women with an interval longer than 2 years (OR = 2.93, 95% CI 1.32–6.48). Age, parity and occupation showed a significant association with POP. Education level and physical work during pregnancy and average birth interval were not associated with POP.

Table 3 presents the results of the multivariable logistic regression analysis for POP where we tested the relationship of short birth interval and POP. Since age and parity were highly correlated, we adjusted for age and occupation in Table 3. After adjusting for age and occupation, the minimum birth interval was significantly associated with POP (AOR = 3.08, 95% CI 1.04–9.19). The likelihood of POP was higher among housewives (AOR = 4.04, 95% CI 1.27–12.8) than among women in the business group. The likelihood of POP was higher among women aged 30–39 years (AOR = 6.77, 95% CI 1.79–25.66) and 40–49 years (AOR = 15.60, 95% CI 3.61–67.41) than those aged 20–29 years.

### Self-reported reasons for POP

Among the 131 participants, 39 had POP. Each of them had their own story about the condition. A 45-year-old woman explained how she was coping with POP as follows: “I felt something was coming out and blocking the opening of the vagina. I felt it was increasing while carrying heavy loads (water, firewood) and while washing or cleaning in a squatting position.”

Out of the 131 women, fifty-four had complications during or after their pregnancies, such as prolonged labor or heavy bleeding with or without retained placenta. A 42-year-old woman described her past experience as follows: “During the first delivery, I faced 5 days and nights of labor pain and I was shifted from the local health post to a primary health care center for further procedures. At that time everything was gone, a total tear of the pelvic floor during the second pregnancy, and I had a hemorrhoid problem. It was so painful.”

Among those with POP, 30 out of 39 women were satisfied with the treatment that they were receiving from public health facilities. The treatments included applying ring pessaries, taking medicines per the advice of medical experts, undergoing surgery, and performing pelvic floor exercises.

Likewise, a doctor said, “As a medical doctor, I don’t think prolapse is a medical problem. If it were, it would have ended with surgical intervention. But women here continue to suffer even after surgery. I think prevention, awareness and removing risk factors can solve the problem in the long run. Although through this camp we will do surgeries for so many women, but there won’t be postsurgical care or, if there is, we cannot guarantee that all the women here who had surgery will be there for the postsurgical care later, and these women have to walk for several hours to return home after this surgery.”

Similarly, most of the women seem to be influenced with patriarchal norm pressure to have a male child and go through repeated pregnancies to do so, resulting many pregnancies without proper birth spacing. A 41-year- old woman says, “I had to do all the household chores and agricultural work along with looking after my in-laws and, five children. 4 daughters and then a son. My husband is working abroad. I couldn’t rest properly after giving birth to each child.”

Referral and curative health services are poor despite improvements in primary and preventive health. However, locals are happy that they can obtain access to at least those limited health resources. It was also discovered that almost all the women from the surrounding area gathered for the free health checkup camp, which was held in Panchapuri municipality in February 2019. Women of different age groups, from the young to the elderly, participated in this forum and discussed their health problems without any reluctance, which was unexpected. They remained aware of reproductive health issues and were happy to share their feelings. The UNFPA reports that 80% of the women who went through surgery for uterine prolapse say they lost hope in life and hide their problems within themselves. However, in this study, women were not shy. Several participants revealed that they got information from female community health volunteers (FCHVs) during mothers’ group meetings, where information was provided to local women on maternal and child health issues.

## Discussion

This study was designed to assess the effect of birth spacing on the prevalence of POP. The findings show that the minimum birth interval was significantly associated with POP. This was the first epidemiological study conducted in Nepal with a focus on short birth interval and prevalence of POP. Although WHO has recommended a healthy pregnancy interval of at least 2 years (24 months), a majority of the women in this study reported the short birth interval of less than 2 years. Similarly, there are many countries reporting high percentages of pregnancies occurring within 24 months: Uttar Pradesh, India (30%) [28], Pakistan (60%) [29], and Kenya (50%) [30]. If a woman has more than one child and the birth interval between the two children is short, it may result in a higher risk of POP. Short interpregnancy interval increases risk for uterine rupture and other major morbidities [18]. Closely spaced and multiple births may have adverse long-term implications for women’s health [12]. Frequent conception, giving birth to many children, and lack of access to skilled attendants are also the main causes of POP [31]. A previous study by Bonetti et al. reported that 18 out of 32 women mentioned frequent childbearing as one of the perceived causes of POP [11].

The findings of this study indicate that POP is highly prevalent among women of reproductive age in Nepal. In this cross-sectional study, the prevalence of POP was found to be 29.8% which is relatively high compared to the previous study in Nepal which was found to be 10% [32], while another questionnaire-based study from Bangladesh reported the prevalence as 15.6% [33]. Even much higher prevalence of 64.6% was found from a recent study in Tanzania [34].

Our findings also showed that the prevalence of POP increases with age. This agrees with previous studies [8, 31, 35]. Notably most of the participants in this study were illiterate or had a lower level of education. Very few of them had a secondary- or college-level education. One of the previous studies reported that illiterate women have almost double the number of childbirths without proper birth spacing in Nepal [16]. In rural Nepal, women perform heavy physical work irrespective of their health condition. Our results agreed with the finding by Chiaffarino et al., which showed that housewives involved in heavy physical work were at increased risk of POP [36].

Nepal has made remarkable progress in improving maternal health. It is currently on track to meet the Sustainable Development Goal number 3 to ensure healthy lives and wellbeing for women. The total fertility rate (TFR) of women aged 15–49 declined from 4.1 in 2000 to 2.3 in 2016 according to the Ministry of Health of Nepal [37]. Even though this progress has been realized, the unique social and cultural practices in some rural areas of Nepal encourage women to bear many children at a young age and in a short span of time. This results in many pregnancies at brief intervals, which may significantly affect maternal health [38, 39]. For example, Nepalese women giving birth to a female child tends to have a shorter birth interval and use a shorter acting contraceptive in order not to wait too long to get a male child [11, 36, 40, 41]. Similarly, women living in rural areas may perform heavy physical work immediately after conceiving, irrespective of their health condition. Early marriage, early pregnancy, unassisted home delivery, lack of health facilities, and an unwillingness to seek health care during pregnancy and short birth interval due to various religious and social taboos are the major contributory factors [16, 32]. If women have so many children within a short interval, they cannot rest and work properly, and their children may have inadequate nutrition. In addition, they cannot give proper care equally to all the children [31].

## Limitations of the study

One study limitation was the small sample size. Due to the limited number of cases, we recommended that similar study should be conducted using a larger sample size. This study was performed in only one municipality in mid-western Nepal, which may limit generalization to the whole of Nepal.

## Conclusion

This study was unique in performing quantitative and qualitative research on the effect of short birth intervals on POP. The short birth interval was significantly associated with POP in a rural area of Nepal. Further studies are required to confirm the association.

## Supplementary Information

**Additional file 1: Supplementary material S1.** Pelvic organ prolapse questionnaire 2019

## Data Availability

The datasets analyzed during the current study are available from the corresponding author on reasonable request.

## Abbreviations

POP: Pelvic organ prolapse
PFD: Pelvic floor disorder
WHO: World Health Organization
COPD: Chronic obstructive pulmonary disease
SPSS: Statistical Package for the Social Sciences
OR: Odd ratios
CI: Confidence interval
OB/GYN: Obstetrician/gynecologist
TFR: Total fertility rate
MOH: Ministry of Heath
VDC: Village development committee
PHCC: Primary health care center
HP: Health post
UNFPA: United Nations Population Fund
VH: Vaginal hysterectomy
FCHVs: Female community health volunteers
RA: Research assistant
