# Supplementary material for: The relationship between pelvic organ prolapse and short birth intervals in a rural area of Nepal
Source: Trop Med Health. 2021 Jan 15;49:5. doi: 10.1186/s41182-021-00298-z (PMC7809744; doi:10.1186/s41182-021-00298-z)
Supplement: Supplementary file 1 — Additional file 1: Supplementary material S1. Pelvic organ prolapse questionnaire 2019 [file 41182_2021_298_MOESM1_ESM.pdf]

1. **Supplement File 1**

PELVIC ORGAN PROLAPSE

QUESTIONNAIRE 2019

SECTION 1 Demographic and Economic Information.

A. HOUSEHOLD FEATURES:

Household I.D	Age	Education Level codes	Occupation -codes-	Ethnicity Codes

Education	Occupation	Ethnicity
1=College/University<br>2=Secondary<br>3=Primary<br>4=Lower class<br>5 = N/A	1= Salaried employee<br>2= Own business<br>3= Casual labourer<br>4= agriculture<br>5=Student<br>6=Housewife<br>7=Unemployed	1= Brahman<br>2= Chhetris<br>3= Dalits<br>4=Others (Specify)

B. What is your monthly income (NRs)?

1. Below 5000 2. 5000 – 10000
3. 1000-15000 4. 15000-20000
5. > 20000

Section II - Questions Related to Prolapse

A. What was your age at marriage?

B. What was your age at the time of first childbirth?

1. Below 15 years 2. 15 – 20 years 3. 20 – 25 years
4. 25– 30 years 5. 30– 35 years 6. 36 years or above

C. How many children do you have and how old are they?

D. What is the interval between two consecutive pregnancy?

1. 1st and 2nd child
2. 2nd and 3rd child
3. 3rd and 4th child
4. 4th and 5th child
5. 5th and more...

E. Where did you deliver your child?

First child 1. home 2. health institution 3. others

Second 1. home 2. health institution 3. others

Third 1. home 2. health institution 3. others

Four or more 1. home 2. health institution 3. Others

F. What type of delivery was?

Number of Deliveries	STATUS	
First	Normal	Complicated (Specify)
Second		
Third		
Fourth		
Five or more (specify)		

G. What was the sex of child?

I. First 1. Male 2. Female

II. Second 1. Male 2. Female

III. Third 1. Male 2. Female

IV. Fourth 1. Male 2. Female

H. Did you used any contraceptive method? If Yes, then which one?

I. After which child did you experience problem? What was the problem?

1. First
2. Second
3. Third
4. Fourth

J. What was the work load (physical work) during pregnancy period?

First child: 1. As usual 2. >than before pregnancy 3. < before pregnancy

Second: 1. As usual 2. >than before pregnancy 3. < before pregnancy

Third: 1. As usual 2. >than before pregnancy 3. < before pregnancy

Fourth or more: 1. As usual 2. >than before pregnancy 3. < before pregnancy

K. When did you resume performing physical work after child birth?

L. Did you have any abortion?

1) Yes 2) No

If yes, who assisted abortion process?

a) Trained health workers b) Traditional healers

c) Self medications d) others

M. Did/do you smoke?

Past status 1. Yes 2. No

Present status of smoking 1. Yes 2. No

If yes, for how many years have you been smoking?

-

N. Do you have long term constipation?

During pregnancy 1. Yes 2. No

Post-natal period 1. Yes 2. No

At present 1. Yes 2. No

O. Did you have any complication or diseases during pregnancy?

1. Yes   2. No

If yes which one? ……………………………………

……………………………………

P. Did you have any diseases after child birth?

1. Yes   2. No

If yes which one? ……………….

Part III Health Services related information:

A. Where do you go for the first time for the treatment of your HR problems?

1. Public Health institutions   2. Traditional healers (Dhami, Jhakri)
3. Private Medical Centre   4. Others

B**. Have you treated against uterine prolapse?**

1. Yes   2. No

If yes, from where you get the service?

1. Home   2. Traditional healers   3. Hospital   4. Private clinic

5.Others (specify)

C. What type of treatment have you got?

1. Apply ring pessary
2. Medication as per the advice of health workers
3. Surgery/operation
4. Pelvic floor exercise
5. Others ……

D. What your health status after receiving treatment?

1. improved   2. not improved   3. worse

E. Are you satisfied with available health services?

1. Yes   2. No

F. how can we prevent the uterine prolapse? (Give your opinion, Tick Yes or No for each statement)

1. Do not lift heavy weight during pregnancy.
2. Do not apply pressure before true labor pain.
3. Eat nutritious diet at the time of pregnancy & delivery.
4. Avoid chronic constipation.
5. Avoid long term coughing.
6. Deliver the baby in health institution by health personnel.
7. Abortion through trained health personnel.
8. Treat diabetes & other chronic diseases in time.
9. Do not give birth too many babies.
